# 尺寸排阻-反相液相色谱-质谱联用技术在大鼠肾脏翻译后修饰蛋白质鉴定中的应用

**DOI:** 10.3724/SP.J.1123.2020.05028

**Published:** 2021-01-08

**Authors:** Jianmin LI, Yue ZHUO, Yida ZHANG, Na LI, Jianlin WU

**Affiliations:** 澳门科技大学中医药学院, 中药质量研究国家重点实验室, 澳门 999078; Faculty of Chinese Medicine, State Key Laboratory of Quality Research in Chinese Medicine, Macau University of Science and Technology, Macau 999078, China; 澳门科技大学中医药学院, 中药质量研究国家重点实验室, 澳门 999078; Faculty of Chinese Medicine, State Key Laboratory of Quality Research in Chinese Medicine, Macau University of Science and Technology, Macau 999078, China; 澳门科技大学中医药学院, 中药质量研究国家重点实验室, 澳门 999078; Faculty of Chinese Medicine, State Key Laboratory of Quality Research in Chinese Medicine, Macau University of Science and Technology, Macau 999078, China; 澳门科技大学中医药学院, 中药质量研究国家重点实验室, 澳门 999078; Faculty of Chinese Medicine, State Key Laboratory of Quality Research in Chinese Medicine, Macau University of Science and Technology, Macau 999078, China; 澳门科技大学中医药学院, 中药质量研究国家重点实验室, 澳门 999078; Faculty of Chinese Medicine, State Key Laboratory of Quality Research in Chinese Medicine, Macau University of Science and Technology, Macau 999078, China

**Keywords:** 蛋白质组学, 尺寸排阻色谱, 翻译后修饰, proteomics, size exclusion chromatography (SEC), post-translational modification (PTM)

## Abstract

LC-MS联用技术在蛋白质组学研究中具有重要的作用,但是在复杂的生物体系中,由于样品的高度复杂性和其中蛋白质含量的巨大差异,执行全面且无倾向的蛋白质组分析是一项挑战。因此,在液相色谱分离中采用基于不同原理的色谱分离方法来降低蛋白质样本的复杂度,并对微量蛋白质进行富集,对后续采用质谱方法进行信息的采集和深入分析至关重要。在这里我们开发了一种基于尺寸排阻色谱(SEC)与反相液相色谱(RPLC)结合的新方法来进行复杂体系蛋白质的分离和鉴定,特别是对于微量蛋白质的分析。首先使用SEC对蛋白质进行分离和富集,并酶解成多肽,再通过RPLC-MS联用的方法对酶解后的多肽进行分离和鉴定。结果显示使用上述方法可以有效降低蛋白质样本的复杂度,并有效提高微量蛋白质的鉴定能力,可从大鼠肾脏鉴定出23621个肽段及1345个蛋白质,比常规的二维强阳离子交换-反相液相色谱法(2D SCX-RPLC)鉴定到的肽段及蛋白质分别多出69%及27%。此外,该方法对肾脏翻译后修饰(PTM)蛋白质的鉴定显示出更多的优势,翻译后修饰的多肽鉴定率显著增加,特别是磷酸化肽段的鉴定效率可达到靶向富集策略的水平。在此展示的SEC-RPLC-MS可以更好地了解蛋白质翻译后修饰对肾脏的影响,最终将有助于增加我们对正常的生理性肾功能以及病理过程机制的理解。

## Abstract (en)

Proteomics is an emerging field that has been shown to play a crucial role in unveiling the mechanisms underlying physiological and pathological processes, and liquid chromatography-mass spectrometry (LC-MS) is one of the most important methods employed in this field. However, in complex biological systems, such as eukaryotes, it is challenging to perform a comprehensive and unbiased proteome analysis due to the high complexity of biological samples and enormous differences in sample contents. For example, post-translational modifications (PTMs) in proteins are imperative for cell signaling, but post-translationally modified proteins account for about 1% of the total proteins in a single cell, making their identification extremely difficult. Therefore, chromatographic separation methods based on different principles are generally applied to reduce the complexity of biological samples and enrich trace proteins for their identification through mass spectrometry (MS). In this study, we developed a new proteomics method by combining size exclusion chromatography (SEC) and reversed-phase chromatography (RPLC), to separate and identify trace proteins in complex systems. SEC was used to separate and enrich kidney-specific proteins. After optimization of the method, it was found that 30 mmol/L of ammonium acetate could efficiently separate rat kidney proteins from the total protein fraction so that they could be eluted based on their relative molecular mass. Sodium dodecyl sulphate-polyacrylamide gel electrophoresis (SDS-PAGE) analysis and LC-MS results showed that our SEC separation method not only refined the protein composition of the biological sample but also enhanced the relative contents of trace proteins through multiple injections. The collected protein fractions were further concentrated through ultrafiltration centrifugation followed by freeze-drying, which further improved the recovery of trace proteins by approximately 90% and largely decreased the time required with the use of freeze-drying alone. Thereafter, five protein fractions were separately digested using trypsin, and the resultant peptides were further analyzed by reverse phase chromatography-MS analysis. In the RPLC column, the peptides were isolated mainly based on their hydrophobicity. As a result, by combining SEC and RPLC, 23621 peptides and 1345 proteins were identified from the kidney, with an increase in numbers by 69% and 27%, respectively, when compared to those obtained using the common 2D strong cation exchange (SCX)-RPLC-MS method. However, no significant difference was observed in the pI and grand average of hydropathicity (GRAVY) values. Gene ontology (GO) analysis revealed an increase in the number of proteins in each cell component, especially the membrane. Furthermore, identification of a higher rate of identified peptides than proteins suggested that the protein coverage was also improved, thereby facilitating the detection of PTM proteins. Consequently, five common PTMs in biological processes, including methylation, acetylation, carbamylation, oxidation, and phosphorylation, were examined and compared between the two methods. As expected, the number of post-translationally modified peptides identified using SEC-RPLC-MS were 1.7-1.9 times more than those determined using the SCX-RPLC-MS method. Especially for the identification of phosphorylated peptides, we could achieve the level of the targeted enrichment strategy; however no significant difference was observed in the extents of phosphorylation among serine, threonine, and tyrosine. These results further indicate that upon combining SEC and RPLC, high efficiency could be achieved by decreasing the complexity of the protein sample, and the identification was unbiased. Finally, the phosphorylation of some kidney proteins, such as spectrin, L-lactate dehydrogenase, and ATPases, was found, which is critical for their functions. In summary, the SEC-RPLC-MS approach was developed for the identification of rat kidney proteins and is especially applicable for the identification of PTM proteins. Using this method, the identification efficiency for PTM peptides increased significantly. Therefore, this method has potential for better understanding the impact of PTM on kidney proteins and further elucidating the potential mechanisms underlying its physiological and pathological functions.

蛋白质组学研究近年来引起了越来越多的关注,并且处于技术发展阶段^[1]^。蛋白质组学的主要挑战是鉴定高度复杂、宽动态范围样品中的低丰度蛋白质,由于样品的复杂性和广泛的动态范围,执行无差别的蛋白质组分析是一项非凡的挑战。传统的一维液相色谱(1D-LC)通常无法为复杂样品的分析提供足够的分离能力^[2,3]^,这激发了不同液相色谱技术(例如二维液相色谱,2D-LC)^[4]^组合的发展。在2D-LC中,收集来自第一维液相色谱(第一液相色谱)的流分,然后将其重新注入第二维液相色谱(第二液相色谱)^[5,6]^,两种不同的分离方法相结合可以获得更高的分离效率^[7]^。如2D强阳离子交换-反相液相色谱法(2D SCX-RPLC),由于SCX色谱柱上的静电相互作用和C18色谱柱上的疏水相互作用相结合,具有较高的检测灵敏度和出色的分离性能,广泛应用于自下而上的鸟枪法蛋白质组学当中^[8,9]^。

尺寸排阻色谱法(SEC)是一种广泛用于蛋白质分离纯化的传统技术,该技术主要用于制备纯化和脱盐,尤其是在分子生物学中,该技术被大量用于生产纯净蛋白质^[10,11,12,13]^。反相液相色谱(RPLC)由于其多功能性、灵活性和耐用性,是分析多肽及蛋白质的主要方法之一^[14]^。肽段进入反相色谱柱后,利用肽段的疏水性,根据不同分配系数依次流出,实现肽段分离。利用SEC对蛋白质进行分离纯化,不仅可以降低样品复杂度,并且可以对复杂蛋白质样品中的低丰度蛋白质进行富集,再结合RPLC进行肽段分离实现肽段分析。

蛋白质的翻译后修饰(PTM)是具有许多生物学功能的细胞蛋白质的重要调控机制之一。蛋白质的翻译后修饰广泛存在于各种细胞器的蛋白质中,并且在不同种类细胞器中具有不同的生物功能^[15]^。随着质谱技术的广泛应用,这些化学修饰在蛋白质质量上的变化能被精确地检测到,并且能进行大规模、全面的PTM筛选,以实现对蛋白质生理研究以及疾病预防治疗更深入的了解。迄今为止,已鉴定出超过450种独特的蛋白质修饰,包括磷酸化、乙酰化、氨基甲酰化、氧化等,它们可以通过翻译后修饰改变目标蛋白质的活性、分布、相互作用及寿命^[16]^。每个细胞当中具有翻译后修饰的蛋白质总量约占单个细胞总蛋白质的1%^[17,18,19,20]^,且蛋白质浓度的差异甚至可以达到数百万至数十亿倍,所以鉴定低浓度蛋白质及该类蛋白质的翻译后修饰需要更灵敏、准确的分离及检测方法。肾脏作为重要的排泄器官,它的基本功能是生成尿液,借以清除体内代谢产物及某些废物、毒物,同时经重吸收功能保留水分及其他有用物质,而PTM参与肾脏中各种细胞过程和信号传导途径^[21,22,23,24,25,26,27]^。最近的研究表明肾脏缝隙隔膜、足细胞、神经节和足突的局部粘着可能存在蛋白质修饰^[22-26,28]^。这些肾脏中的蛋白质修饰,不仅在肾小球超滤功能中,而且在肾稳态调节过程中也起着关键作用。而磷酸化修饰作为最普遍的PTM,几乎与每个细胞过程密切相关^[29]^,它在调节多种代谢酶的活性中起关键作用^[30]^,如协调激活ATP合酶为细胞提供能量维持细胞正常运作等^[31]^。

为了鉴定更多的大鼠肾脏蛋白质以及翻译后修饰的蛋白质,我们构建了SEC-RPLC-MS系统,采用SEC对肾脏蛋白质样品进行预分离,降低样品蛋白质复杂度,并使用nano-RPLC-MS对预分离蛋白质的胰蛋白酶解肽进行分离和鉴定。

## 1 实验部分

### 1.1 材料与仪器

醋酸铵、磷酸化盐、乙腈(均来自美国Sigma-Aldrich公司);二硫苏糖醇(DTT),碘乙酰胺(IAA)和尿素(来自美国GE Healthcare公司)。胰蛋白酶(质谱纯)(来自美国Thermo Fisher Scientific)。Dionex Ultimate 3000 RSLC UPLC & Bruker maXis Impact Q-TOF-MS串联质谱;ST-16R型高速冷冻离心机(美国Thermo公司);水由Milli-Q Ultrapure水系统(美国Millipore公司)生产。

### 1.2 样品制备

实验大鼠是购自香港中文大学的Sprague-Dawley (SD)大鼠,并在澳门科技大学的动物房中繁殖。选用8周龄的雄性SD大鼠(225 g),麻醉后生理盐水腹腔主静脉灌注洗净血液,取出肾脏用磷酸缓冲盐溶液(PBS)洗净。横向切取右肾组织100 mg,加入1 mL PBS匀浆,离心后取上清液。300 μL上清液加入1200 μL冷丙酮沉淀,6000 r/min下离心5 min,取蛋白质沉淀。蛋白质沉淀加入300 μL PBS复溶,12000 r/min下离心10 min后取290 μL上清液待用。

### 1.3 肾脏蛋白质SEC预分离

收集肾蛋白质上清液采用BCA(bicinchoninic acid)方法测定蛋白质浓度后,使用Agilent 1100 system制备液相系统将290 μL (827.5 μg)肾蛋白质溶液进行SEC预分离。对于SEC,使用Phenomenex(美国Torrance公司)的BioSep-SEC-3000色谱柱(300 mm×7.8 mm)、Cartridges GFC 3000保护柱(3.0 mm×4 mm)。流动相为30 mmol/L醋酸铵(pH值调至7),流速为1 mL/min,在280 nm处进行吸光度检测,依次收集蛋白质流分。回收的蛋白质流分使用Millipore超滤离心管(3 K, 15 mL)在4000 g下离心浓缩45 min,转移浓缩液至离心管内。冷冻后,使用冷冻离心干燥机除去剩余的液体,收集蛋白质粉末。

### 1.4 蛋白质酶解

收集的预分离肾脏蛋白质粉末加入100 μL 8 mol/L尿素进行复溶,12000 r/min下离心5 min后取上清液,使用BCA方法测定蛋白质浓度。取总蛋白质质量为100 μg的上清,在室温下用DTT(200 mmol/L)还原60 min。加入IAA(1 mol/L),转移至黑暗中孵育60 min。反应混合物在室温下用DTT(200 mmol/L)淬灭60 min。加入25 mmol/L的碳酸铵溶液,直到尿素浓度低于1 mol/L。最后,加入2 μg胰蛋白酶,在37 ℃烘箱消化过夜。用SPE C18柱实现消化液除盐,洗脱液在氮气下干燥。

### 1.5 nano-RPLC-Q-TOF-MS

用50 μL 2%乙腈(含0.1%三氟乙酸, v/v)溶解胰蛋白酶解肽,12000 r/min下离心10 min后取上清液进样。使用Ultimate 3000 RSLC UPLC(Dionex)与maXis Impact Q-TOF-MS(Bruker)串联系统实现肽段分离和鉴定,正离子模式使用Bruker Advance CaptiveSpray离子源。上清液上样到Acclaim PepMap 100 nanoViper C18捕获柱(2 cm×75 μm, 3 μm, Thermo)。然后在Acclaim PepMap RSLC nanoViper C18分析柱(15 cm×75 μm, 2 μm, Thermo)上以300 nL/min的流速分离肽段。流动相由(A)水中含0.1%甲酸(FA)和(B)乙腈中含0.1%FA组成,梯度如下:0~8.0 min, 5%B; 8.0~110.0 min, B从5%线性增加到40%; 110.0~110.1 min, B从40%线性增加到80%; 110.1~115.0 min, 80%B; 115.0~115.1 min,恢复至5%B; 115.1~120.0 min, 5%B。进样量为1 μL。质谱用正离子模式下的Bruker Advance CaptiveSpray离子源。干燥气的温度为160 ℃,流速为4.0 L/min。端板偏移和毛细管电压分别设置为500 V和1400 V。在*m/z* 330和1400之间的范围内选择了前10个前体离子,并且排除了带单电荷的离子,而MS/MS分析首选带电状态为+2、+3和+4的前体离子。

### 1.6 蛋白质鉴定

使用Data Analysis软件处理MS数据导出的. mgf文件,将该. mgf数据导入MASCOT搜索引擎中,数据库为Swiss-Prot 51.6。选择胰蛋白酶作为酶;固定修饰为carbamidomethyl (C),可变修饰为Phospho-S、Phospho-T、Phospho-Y、Acetyl-K、Carbamyl-K、Methyl-DE、Oxidation-M等7个修饰;前体离子和MS/MS允许误差范围分别设定为30×10^-6^(30 ppm)和0.2 Da;肽电荷为2^+^、3^+^和4^+^。

## 2 结果与讨论

我们了构建SEC-RPLC系统,采用SEC对肾脏蛋白质样品进行预分离,降低样品蛋白质复杂度,从而提高低丰度蛋白质的检测灵敏度。

### 2.1 SEC流动相优化及流分浓缩

2.1.1 SEC流动相优化

为提高尺寸排阻色谱对蛋白质的分离能力,首先对流动相进行了优化。20 μL肾脏蛋白质溶液(2.85 μg/μL)进样,分别使用30 mmol/L醋酸铵、10 mmol/L PBS或Milli-Q水作为流动相进行分离,检测波长为280 nm。在尺寸排阻色谱柱中,蛋白质是根据其在流动相中流体动力学体积的差异,通过扩散进出停滞的孔而分离出来的。具有中等离子强度的非变性水性缓冲液(如醋酸铵、磷酸缓冲盐溶液等),通常用于最大程度减少蛋白质与固定相表面的非特异性相互作用^[32,33]^。PBS因能有效提高蛋白质在SEC的分离效率而被广泛应用^[34]^,但缺点是非挥发性盐的残留在后续的质谱检测时会导致质谱损伤;醋酸铵作为挥发性盐被广泛应用到LC-MS串联质谱上,可在提高SEC蛋白质分离效率的同时保证蛋白质结构及活性^[35]^,且作为挥发性盐可避免对仪器的损伤。我们的结果表明,使用醋酸铵的液相色谱图可显示更多的单独色谱峰,峰形更完整,具有更高的分离效率(见图1a),故采用质谱兼容的醋酸铵作为流动相盐。

**图 1:**
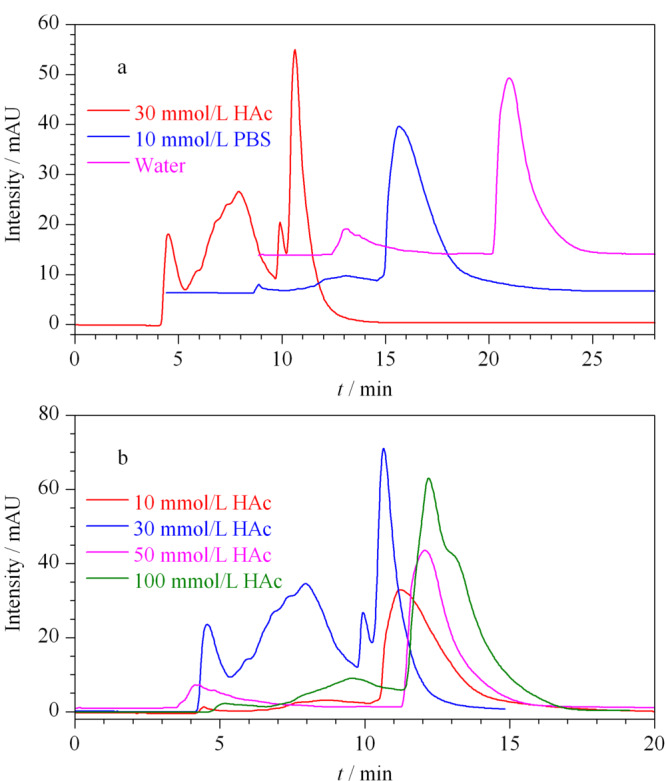
尺寸排阻色谱流动相的优化

接下来,对流动相的盐浓度进行了优化,醋酸铵浓度分别为10、30和50 mmol/L。结果显示30 mmol/L醋酸铵分离效率高于10 mmol/L醋酸铵,提示适当提高盐浓度可减少蛋白质表面的电荷数量,减少蛋白质与填料表面可能的吸附^[36]^。50 mmol/L的分离效果却低于30 mmol/L醋酸铵的分离效果(见图1b)。虽然缓冲盐能抑制蛋白质与填料之间的静电效应,但提高盐浓度也会增强蛋白质与色谱柱填料的疏水相互作用(通常在高盐浓度下占主导地位),影响蛋白质分离效果^[36,37]^。为了最佳的分离效率,及减少盐浓度便于后续实验处理,采用30 mmol/L醋酸铵作为流动相。

2.1.2 蛋白质流分的浓缩

在SEC分离肾脏蛋白质前及浓缩富集后,分别测定蛋白质的量并计算回收率。常规的流分浓缩方法是收集流分后进行冷冻干燥,回收率仅为75.74%,其可能由于样品较大的体积,在处理过程中与容器壁接触面积较大导致样品损失严重;并且该方法所需时间过长,每次冷冻干燥需要20 h以上。为了降低蛋白质损失及减少所需时间,先使用超滤离心管(3 K)对样品进行浓缩,将浓缩的蛋白质溶液进行冷冻干燥,可将浓缩时间降至6~7 h。使用超滤离心管-冷冻干燥方法的蛋白质回收率可以达到89.67%,高于仅使用冷冻干燥浓缩的方法(75.74%)。后续实验均采用超滤离心管-冷冻干燥的方法进行流分浓缩。

### 2.2 SEC-RPLC方法

2.2.1 样品复杂度分析

组织蛋白质组成较血清、细胞蛋白质更为复杂,在当前的研究中,我们测试了使用SEC对组织来源的蛋白质进行预分流来降低样品复杂性的效率。

为了确定尺寸排阻色谱的分离效果,经过SEC分离的蛋白质流分用尿素复溶后,进行SDS-PAGE(sodium dodecyl sulphate-polyacrylamide gel electrophoresis)分析。通过SDS-PAGE(见图2a)发现5个流分中的蛋白质相对分子质量分布呈阶梯状下降,表明使用SEC可通过蛋白质相对分子质量大小进行分离,从而有效降低组织蛋白质样品的复杂度,且多次进样分离,可对低丰度蛋白质进行富集,有效提高低丰度蛋白质的含量。经过RPLC-MS分析后,对各个流分中鉴定的蛋白质相对分子质量分布进行分析,可发现蛋白质相对分子质量分布范围与SDS-PAGE结果相似,呈阶梯状下降,且各流分中蛋白质相对分子质量的中位数亦呈梯度下降(50、818~39、963 Da)(见图2b)。这说明SEC可降低蛋白质样品复杂度,多次进样收集流分可有效富集低丰度蛋白质。

**图 2:**
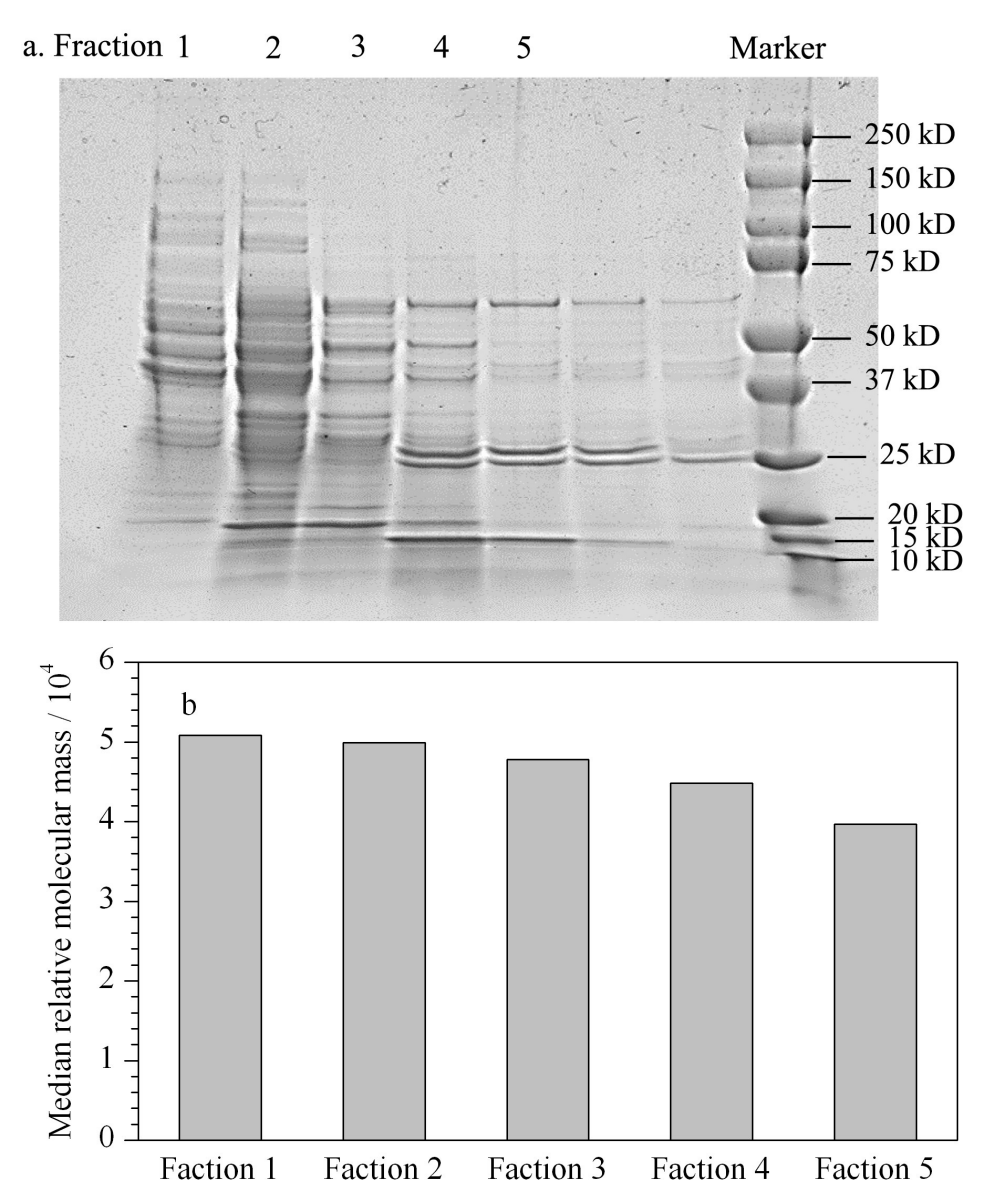
各流分蛋白质的相对分子质量分布

2.2.2 SEC-RPLC提高蛋白质及肽段鉴定效率

如何在高度复杂的宽动态范围样品中鉴定低丰度蛋白质是当今蛋白质组学的主要挑战,在这种宽动态范围下,高丰度蛋白质与低丰度蛋白质的浓度差异极大,高丰度蛋白质的酶解肽段广泛存在,导致该类肽段过度采集,最终使得低丰度蛋白质的肽段信号被掩盖或忽略。

本文所建立的SEC-RPLC-MS方法与平行实验的SCX-RPLC-MS方法^[38]^进行比较,可以发现SEC-RPLC方法中的色谱图响应高达2×10^7^而SCX-RPLC仅能达到2×10^6^。将各流分蛋白质数据合并重复项并去除冗余后,SEC-RPLC鉴定出1345个蛋白质和23621个肽段;SCX-RPLC可鉴定出1053个蛋白质和13935个肽段,即SEC-RPLC较SCX-RPLC可显著提升肽段鉴定数量(1.69倍)及蛋白质鉴定数量(1.28倍)(见图3)。

**图 3:**
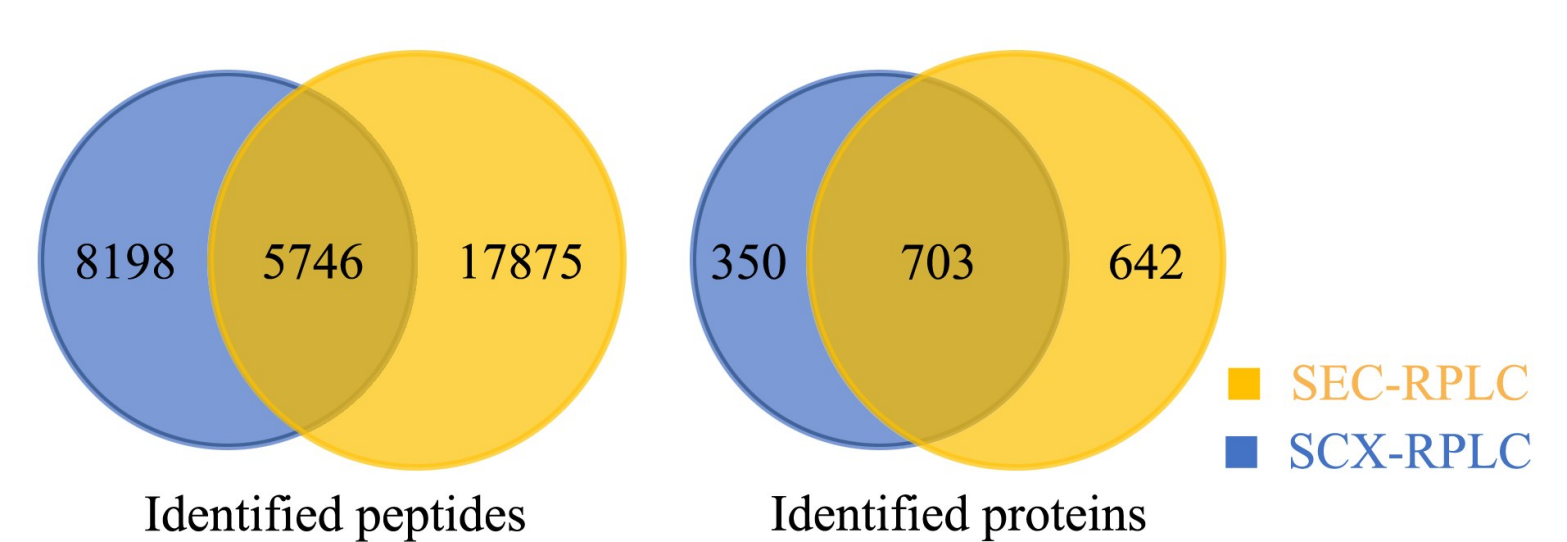
SEC-RPLC和SCX-RPLC鉴定的蛋白质及肽段数量

对蛋白质及肽段的pI值及GRAVY (grand average of hydropathicity)值进行比较(见图4a, 4b),在SCX-RPLC与SEC-RPLC方法之间均无显著性差异,其符合SEC通过蛋白质大小进行分离蛋白质的物理性质而无倾向性。

**图 4:**
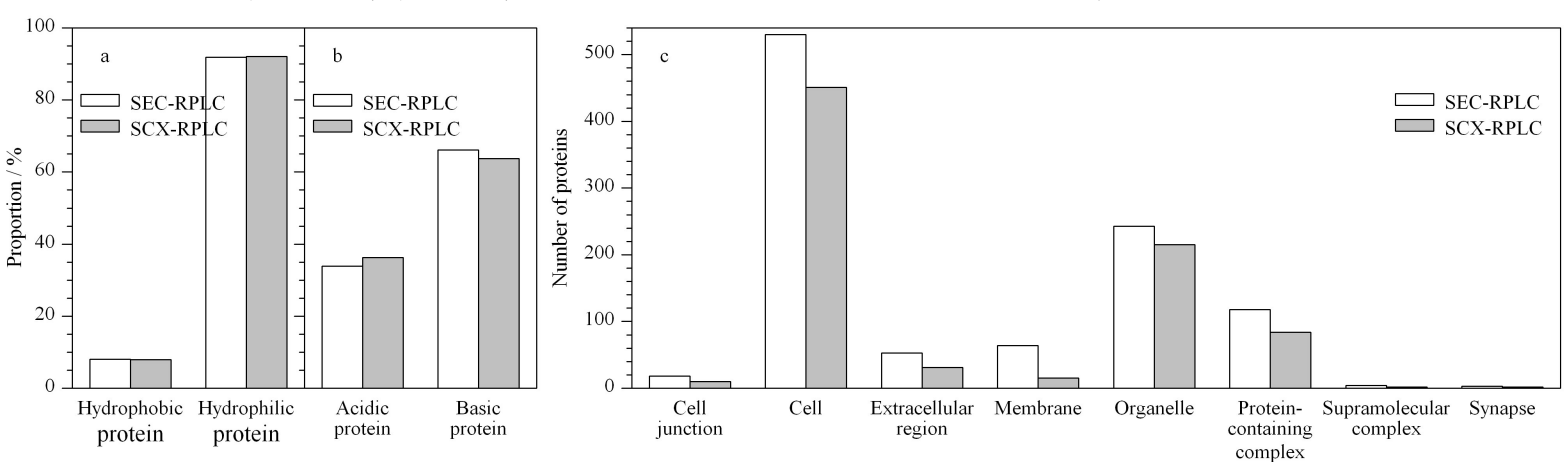
SEC-RPLC和SCX-RPLC鉴定蛋白质及肽段的物理性质及细胞成分分析

对蛋白质鉴定结果进行蛋白质GO(gene ontology)分析及分类,分析结果见图4c, SEC-RPLC各细胞成分蛋白质鉴定数均高于SCX-RPLC方法。通过降低蛋白质样品的复杂度,减少质谱对高丰度蛋白质肽段的过度采集,提高低丰度蛋白质的浓度,最终增加细胞各成分低丰度蛋白质肽段鉴定的可能性。其中,膜蛋白质鉴定数有显著提高(SEC-RPLC: 64; SCX-RPLC: 15);虽然该方法在膜蛋白质的鉴定方面较商业专用试剂盒的鉴定数量(800~1000)仍有较大差距,但商用试剂盒及传统膜蛋白质提取方法需要1 g以上的样品,且多需使用专属的去污剂对膜蛋白进行提取,导致非膜蛋白质变性,无法与膜蛋白质共同检测^[39,40]^。

2.2.3 SEC-RPLC对PTM修饰蛋白质的鉴定

蛋白质的翻译后修饰参与调控多种蛋白质的生物学功能,但翻译后修饰的蛋白质量仅占单个细胞蛋白质总量的1%,使其难以被鉴定^[17,18,19]^。SEC-RPLC-MS方法鉴定的肽段数量较SCX-RPLC-MS方法提升程度显著,但蛋白质鉴定数增加相对较少,说明该方法可以提高蛋白质鉴定的覆盖率,进而可能使更多潜在的翻译后修饰位点被检测,从而提高翻译后修饰被检测的可能性。

为了确定SEC预分离技术鉴定潜在PTM蛋白质的能力,我们比较了使用SEC-RPLC及SCX-RPLC分析的同一组肾脏蛋白质复溶液。如图5所示,在没有预分离的情况下直接进行分析,SCX-RP方法鉴定出9136个PTM肽段,而SEC-RP方法鉴定出17656个修饰肽段,提高了近一倍。

**图 5:**
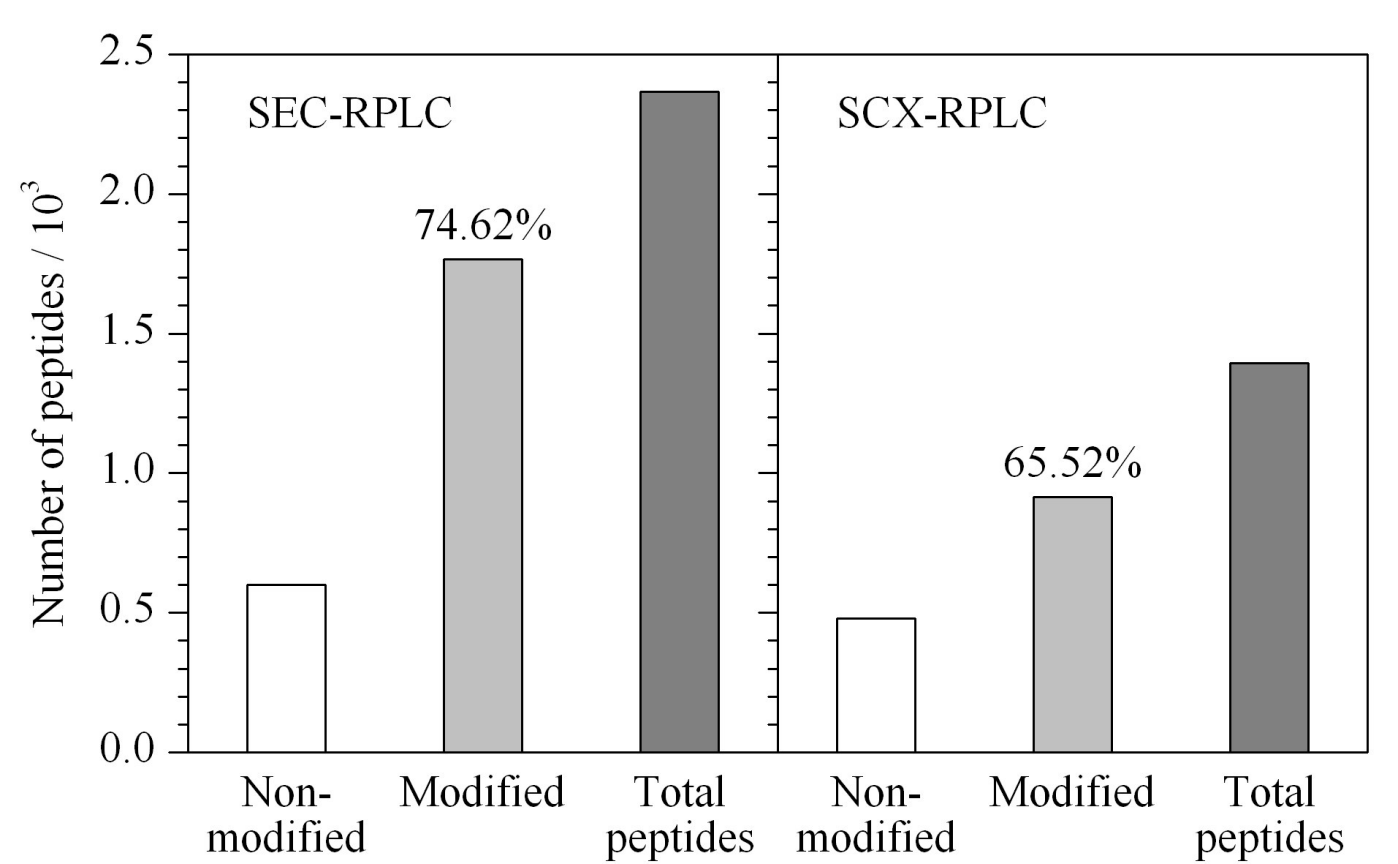
SEC-RPLC和SCX-RPLC鉴定到的总肽段、修饰肽段和未修饰肽段的比较

5种常见翻译后修饰的比较显示(见图6b),使用SEC-RPLC方法鉴定的最多的修饰是甲基化修饰肽段为9599个,是SCX-RPLC鉴定数目的1.9倍;乙酰化修饰肽段鉴定5430个,是SCX-RPLC鉴定数目的1.9倍;氨基甲酰化修饰肽段鉴定3553个,是SCX-RPLC鉴定数目的1.7倍。位于蛋氨酸的氧化修饰肽段鉴定3636个,是SCX-RPLC鉴定数目的1.9倍。

**图 6:**
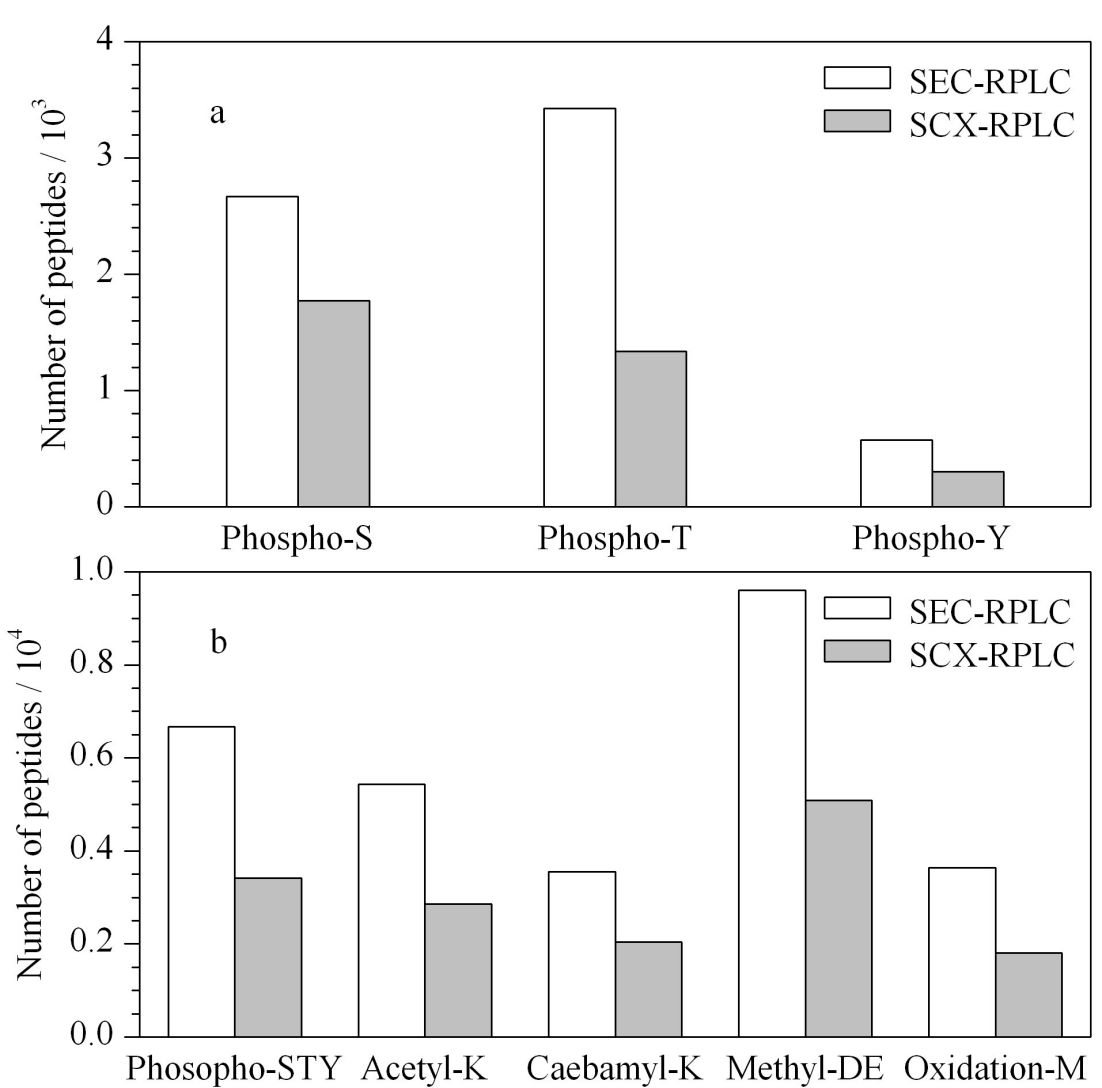
SEC-RPLC和SCX-RPLC鉴定的大鼠肾脏翻译后修饰肽段

与未分离样品(含磷酸化修饰的蛋白质838个)相比,进行预分离的样品鉴定到的含磷酸化修饰的蛋白质数(1171个)是SCX-RPLC鉴定数目的1.4倍(见图6a)。先前的研究^[41,42]^表明,使用SCX或ERLIC(electrostatic repulsion-hydrophilic interaction chromatography)进行磷酸化修饰肽分级分离,然后进行IMAC(immobilized metal ion affinity chromatography)或TiO_2_纯化,可以富集6000多种磷酸化修饰肽段。在这里我们在丝氨酸、苏氨酸或酪氨酸上鉴定了磷酸化修饰的肽段共6665个,而在未分离的蛋白质样品中仅鉴定到3415个磷酸化修饰肽段,增长近2倍。且丝氨酸、苏氨酸、酪氨酸上修饰的肽段鉴定比例无显著倾向性,这种改进可能是由于降低了蛋白质样品的复杂性,从而提高了在MS级别上的鉴定效率。我们的结果表明,SEC预分离与富集策略相结合,是鉴定蛋白质修饰的有力方法,且对于不同的PTM修饰均有作用。

2.2.4 主要肾脏蛋白质的翻译后修饰研究

该项研究中,我们发现的多种被修饰的蛋白质参与了肾脏关键生理功能的调节。我们将以3个磷酸化修饰蛋白质为例进行介绍,它们是磷酸化的血影蛋白(SPTN1_RAT), L-乳酸脱氢酶A链(LDHA_RAT)和过渡性内质网ATP酶(TERA_RAT)。如图7所示,血影蛋白的修饰肽段^653^MNEVISLWK^661^的母离子双电荷峰为[M+2H]^2+^*m/z*600.2724,比相应未修饰的肽段多79 Da,其中碎片离子*b*_1_*~b*_7_、*y*_1_未被修饰,*y*_6_*~y*_8_的碎片离子相对分子质量增加了79 Da,结合二级质谱推断被修饰的位点为S658(见图7a)。L-乳酸脱氢酶A链的修饰肽段^318^KSADTLWGIQK^328^的母离子双电荷峰为[M+2H]^2+^*m/z* 670.84800,比相应未修饰的肽段多94 Da,肽段碎片*b*_1_*~b*_3_相对分子质量未增加,*b*_4_相对分子质量增加了14 Da,可推测在D321上存在甲基化修饰,剩余相对分子质量增加了79 Da,可推测存在磷酸化修饰,通过二级质谱比对肽段碎片*b*_1_*~b*_3_、*y*_1_、*y*_3_相对分子质量未增加,修饰肽段碎片*b*_7_、*y*_8_*~y*_9_的相对分子质量均增加了94 Da,由此进一步推断其被磷酸化修饰的位点为T322(见图7b)。

**图 7:**
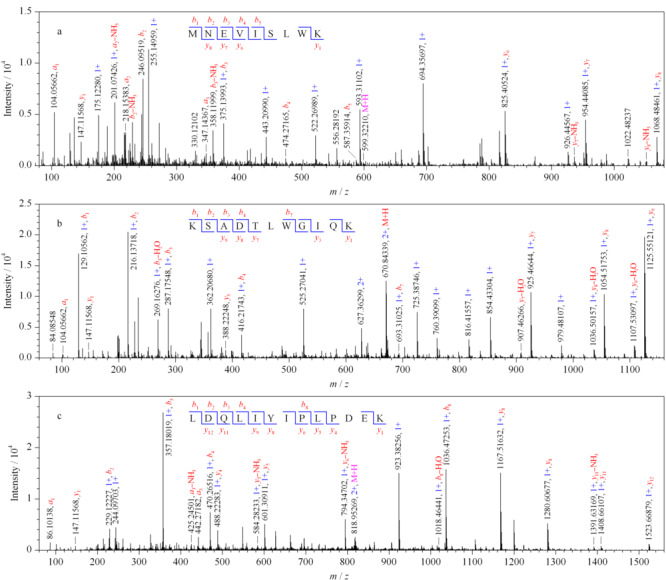
磷酸化修饰肽段二级质谱图

过渡性内质网ATP酶的修饰肽段^639^LDQLIYIPLPDEK^651^的母离子双电荷峰为[M+2H]^2+^*m/z* 818.89579,比相应未修饰肽段多79 Da,其中*b*_1_*~b*_4_、*y*_1_、*y*_4_*~y*_6_未修饰,修饰肽段*y*_8_*~y*_12_的碎片离子相对分子质量增加了79 Da,推断磷酸化修饰位点为Y644(见图7c)。

血影蛋白是肾素多蛋白复合物的重要组成部分,在肾脏细胞中起到调节膜蛋白质及细胞完整性的作用^[43]^; L-乳酸脱氢酶A链参与丙酮酸合成(*S*)-乳酸途径,从而影响肾脏细胞中ATP含量及能量供给^[44]^;过渡性内质网ATP酶为囊泡的形成提供ATP^[45]^。这些蛋白质经磷酸化后显示出生理活性^[43,44]^,且参与到肾脏的关键生理活性及功能中。除磷酸化修饰蛋白质外,我们也鉴定出乙酰化、氨基甲酰化、甲基化、氧化等4种蛋白质修饰,已有相关文献^[46,47,48,49]^表明以上修饰与蛋白质的代谢及稳定性有关,氧化修饰更可能导致蛋白质活性大变,并参与某些疾病的发展。利用SEC-RPLC-MS对蛋白质翻译后修饰进行研究,可鉴定到参与肾脏关键生理活性及功能的蛋白质及修饰肽段,有利于更好地了解蛋白质修饰,并有助于我们了解肾脏蛋白质的正常生理功能及病理机理。

## 3 结论

我们的研究提出了SEC-RPLC-MS二维分离系统,以鉴定大鼠肾脏蛋白质中的微量蛋白质及翻译后修饰的蛋白质。此处报告的数据表明,SEC-RPLC二维分离系统可显著增强肾脏蛋白质及肽段的鉴定,提高了蛋白质覆盖率。我们还展示了通过SEC-RPLC预分离技术提高PTM鉴定效率的可行性,特别是对磷酸化肽段的检测。此方法可应用于大鼠肾脏蛋白质组分析,更好地了解蛋白质修饰,并最终有助于我们解释这些修饰在肾脏的正常生理功能维持和疾病发生中的潜在作用。
